# Finite element analysis of anterior cruciate ligament reconstruction techniques: A comparison of the mechanical properties of all-inside fixation and traditional fixation

**DOI:** 10.3389/fbioe.2024.1438839

**Published:** 2024-08-02

**Authors:** Xiaodong Chen, Changguo Xue, Kuanxin Li, Kecheng Mu, Cheng Yao, Zhiyan Wang, Hongzhi Chen, Jun Zhang

**Affiliations:** ^1^ Anhui Key Laboratory of Tissue Transformation, Department of Orthopedics, The First Affiliated Hospital of Bengbu Medical University, Bengbu Medical University, Bengbu, Anhui, China; ^2^ School of Material Science and Engineering, Anhui University of Science and Technology, Huainan, China; ^3^ Department of Orthopedics, The Third People’s Hospital of Bengbu, Bengbu, China; ^4^ Department of Radiology, The First Affiliated Hospital of Bengbu Medical University, Bengbu, Anhui, China; ^5^ Department of Orthopedics, The First People’s Hospital of Bengbu, Bengbu, China

**Keywords:** anterior cruciate ligament reconstruction, all-inside fixation technique, traditional fixation technique, finite element analysis, knee

## Abstract

**Objective:**

The main purpose of this study was to explore the mechanical properties of the anterior cruciate ligament and its attachments following reconstruction with the all-inside technique after anterior cruciate ligament injury.

**Methods:**

Knee joint computed tomography data were collected from healthy volunteers, and knee joint models were created using Mimics software. A normal knee joint model, an all-inside reconstructed anterior cruciate ligament model, and a traditional reconstructed anterior cruciate ligament model were established. A tensile force of 134 N and a bending moment of 5 N/m were applied at the anterior aspect of the proximal tibia in these three models. The knee joint was subjected to external rotation, internal rotation, varus, valgus, flexion, and extension under this bending moment. The magnitude and distribution of stress on the ligament or graft and the magnitude and distribution of stress on the graft attachments were observed under different loading conditions.

**Results:**

Under different external forces, the maximum stress on the ligament in the normal model fluctuated from 1.949 to 18.302 MPa, with an uncertain distribution of maximum stress. The maximum stress on the graft in the all-inside reconstructed anterior cruciate ligament model fluctuated from 0.705 to 3.465 MPa and was mainly distributed at the junction of the graft and the tibial footprint. In the traditional reconstructed anterior cruciate ligament model, the maximum stress on the graft fluctuated from 5.012 to 59.269 MPa and was primarily distributed at the junction of the interference screw and the graft. The concentration of stress on the loop and plate in the all-inside reconstructed anterior cruciate ligament model fluctuated from 70.461 to 346.363 MPa, with maximum stress distributed at the junction of the loop and the tibial surface. The maximum stress on the interference screw in the traditional reconstructed anterior cruciate ligament model fluctuated from 10.184 to 92.298 MPa, with maximum stress primarily distributed at the end of the interference screw.

**Conclusion:**

Under different external forces, the graft used in all-inside anterior cruciate ligament reconstruction is subjected to fewer external forces than that used in traditional anterior cruciate ligament reconstruction, which may indicate a relatively stable mechanical environment. The strength of the loop and plate can theoretically tolerate daily knee joint movements of patients without injury.

## Background

Anterior cruciate ligament (ACL) injury is a knee joint condition commonly detected in athletes and nonathletes (Lowenstein et al., 2023), occurring in 91–152 individuals per 100,000 people (Tang et al., 2024). Damage to the anterior cruciate ligament can reduce the stability of the knee joint and accelerate the onset and progression of osteoarthritis (He et al., 2022). Traditional reconstruction of the ACL typically involves autograft tendon transplantation, but there is a risk of postoperative ligament laxity or reinjury. Currently, there are two main techniques for reconstructing the ACL: traditional reconstruction and all-inside reconstruction (Xu et al., 2024).

The main difference between all-inside and traditional reconstruction of the ACL involves the tibial end. The all-inside technique primarily involves drilling tunnels in the tibia. Lubowitz (Lubowitz, 2012; Ren et al., 2022) began flipping the flipcutter in reverse to create two tibial bone tunnels with different diameters—the outer of which had a smaller diameter and penetrated the tibial cortical bone and the inner of which had a larger diameter and led to the joint. The ligament on the tibial surface is mainly suspended and fixed by a tape plate (Xu et al., 2024).

The aim of this study was to compare the differences in stress on the grafts and attachments (loop, plate, and interference screw) between all-inside and traditional ACL reconstruction methods using finite element analysis to ultimately explore the potential of the all-inside technique for reconstructing the ACL of the knee joint. This provides a preliminary scientific basis for the clinical selection of the most suitable reconstruction method.

## Methods

### Simulation

A 26-year-old healthy male with a height of 170 cm volunteered to participate in the study and signed an informed consent form. The volunteer’s right knee joint was scanned using a high-resolution computed tomography (CT) scanner, and the CT data were subsequently exported in a DICOM format for further use. Three models were used for reconstruction in this study, namely, the normal model, the all-inside ACL reconstruction model, and the traditional ACL reconstruction model.

Mimics 21.0 (Materialise, Belgium) software was utilized for three-dimensional reconstruction of the DICOM format CT data, with a focus on modelling the distal femur, proximal tibia, and fibula. The bone models were exported in the STL format, imported into Geomagic 2021.0 (3D Systems, United States) for fitting nonuniform rational B-spline (NURBS) surfaces, and then exported in the IGS format. Additionally, using Mimics and 3-Matic, its subsidiary software, models of the meniscus, tibial cartilage, and distal femur cartilage were constructed based on models of the distal femur, proximal tibia, and fibula.

The 2021 version of SolidWorks (Dassault Systèmes, United States) was used to reconstruct the medial and lateral collateral ligaments and the posterior cruciate ligament based on models of the distal femur, proximal tibia, and fibula. The solid cut feature in SolidWorks was used to establish channels for the ligaments at the femoral and tibial ends.

Screws and plates for femoral fixation of ligaments were not included in the modelling process in this study, but interference screws for traditional tibial reconstruction and loops and plates for all-inside fixation were included in the modelling process. Finally, all the designed models were imported into HyperMesh 13.0 (Altair Engineering, United States) for meshing. The mesh models were exported in the inp format and imported into ABAQUS 2021.0 (Siemens, France) for analysis.

#### Material properties

All models in this study were set with isotropic and linear material properties using C3D4. The graft used in this study was similar to a tendon autograft used for ACL reconstruction. The material parameters for this study were determined based on research by Ren (Ren et al., 2022), Zainal Abidin (Zainal et al., 2021), and Marquet-Rivera (Marquet-Rivera et al., 2021), as shown in Table 1.

**TABLE 1:** Material parameters for the models constructed in this study.

Tissue	Young’s modulus (MPa)	Poisson’s ratio
Cortical bone	15, 000	0.32
Trabecular bone	100	0.3
Cartilage	20	0.45
Meniscus	55	0.3
ACL and graft	64	0.45
Posterior cruciate ligament	67	0.45
Medial and lateral collateral ligaments	61	0.45
Interference screw	1, 200	0.33
Plate	110, 000	0.3
Loop	12, 500	0.3

Note: ACL, anterior cruciate ligament.

#### Contact properties

In this study, the contact properties between the femoral cartilage and meniscus were set as surface-to-surface contact, with tangent behavior simulated as hard contact, and frictional contact with a friction coefficient of 0.1 (Bae et al., 2016; Kim et al., 2021). The interaction between the meniscus and the tibial cartilage was set as tied; the medial and lateral collateral ligaments and the posterior cruciate ligament with the tibia and femur were set as tied at the contact points, simulating their original physiological properties.

The graft with the femoral and tibial channels was set as tied because it is immobile under physiological conditions. The boundary condition for the graft at the femoral end was set to fix its six degrees of freedom. The interaction between the graft end in the traditional reconstruction model and the interference screw was set as tied.

The graft used for all-inside reconstruction was connected to the loop at the tibial end, suspended and fixed with the loop and plate on the tibial end, and then tied together, thus setting the interaction among the graft, the loop, and the plate as tied. The plate with the tibial surface was set as tied because it is fixed in clinical practice.

#### Boundary conditions

**FIGURE 1:**
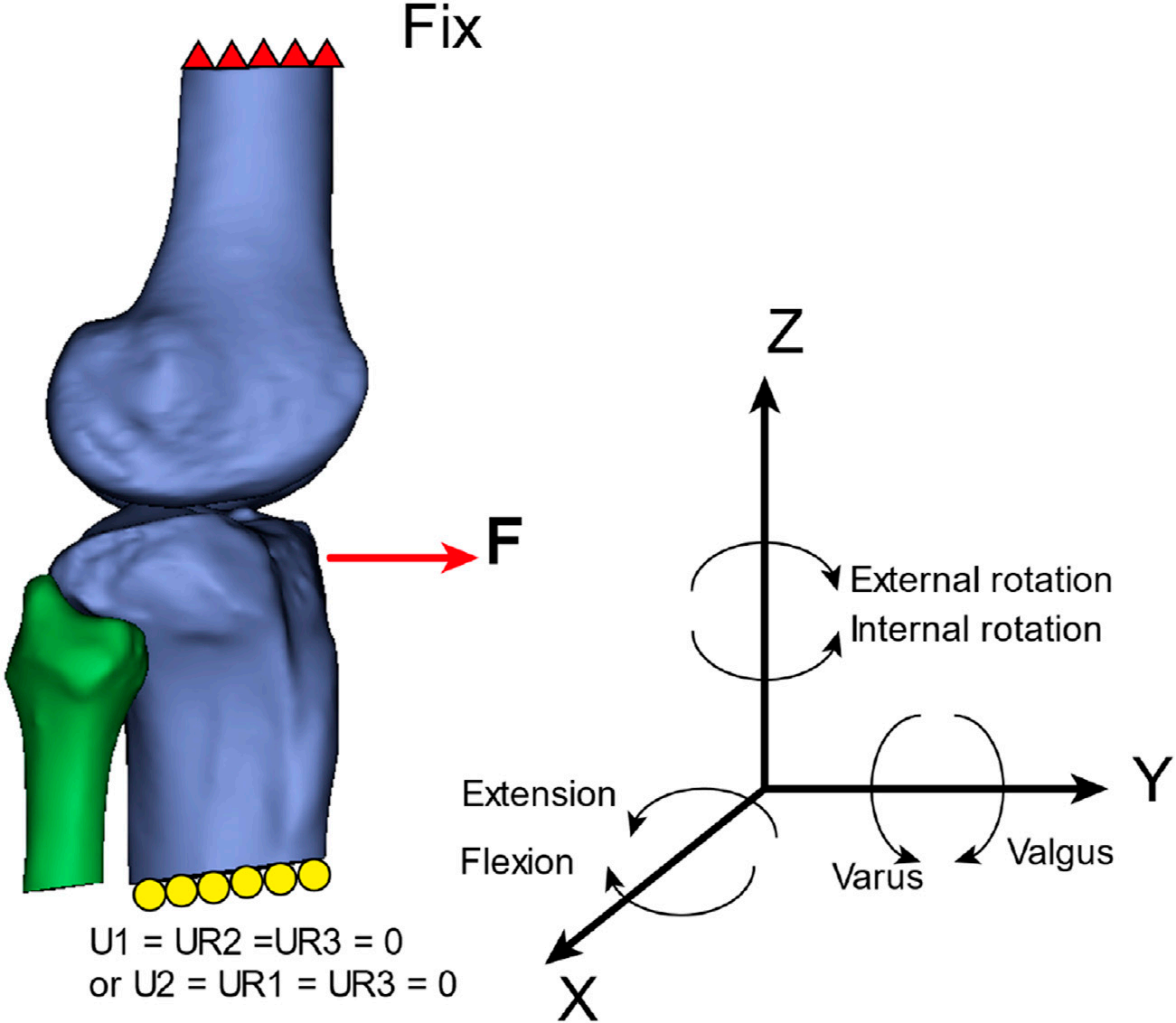
External load application method and boundary conditions.

The boundary conditions for the models in this study included fixing six degrees of freedom at the upper end of the femur. At the lower end of the tibia in the global coordinate system of this study, the boundary conditions were set as follows: the translational degrees of freedom along the X-axis (U1) and the rotational degrees of freedom around the Y-axis and Z-axis (UR2 and UR3) were set to zero, or the translational degrees of freedom along the Y-axis (U2) and the rotational degrees of freedom around the X-axis and Z-axis (UR1 and UR3) were set to zero (refer to Figure 1 for an illustration).

#### Load application

External load application (Zainal et al., 2021).

1. A forward load of 134 N was applied at the proximal tibia to simulate knee joint stability. 2. A torque of 5 N/m was applied to simulate external rotation of the lower leg. 3. A torque of 5 N/m was applied to simulate internal rotation of the lower leg. 4. A torque of 5 N/m was applied to simulate varus movement of the lower leg. 5. A torque of 5 N/m was applied to simulate valgus movement of the lower leg. 6. A torque of 5 N/m was applied to simulate knee flexion. 7. A torque of 5 N/m was applied to simulate knee extension.

The magnitude of stress and the location of maximal stress distribution at the anterior cruciate ligament or graft and its attachments under different loading conditions in the three models were assessed in this study (refer to Figure 1 for an illustration).

### Statistical analysis

The Mann–Whitney U test was performed to analyze the results of each group. Continuous variables are expressed as the mean and standard deviation. The analysis was conducted using Python (version 3.8) in Jupyter Notebook, and a *P*-value less than 0.05 indicated a statistically significant difference.

## Results

Three models were established in this study: the normal knee joint model, the all-inside ACL reconstruction model, and the traditional ACL reconstruction model. Each model included the ACL or graft, posterior cruciate ligament, femoral cartilage, tibial cartilage, medial and lateral menisci, and medial and lateral collateral ligaments. The interference screw, the loop, and the plate were also attached in the models used for reconstructing the ACL. The diameters of the graft and the tunnels in the femur and tibia were 8 mm.

**FIGURE 2:**
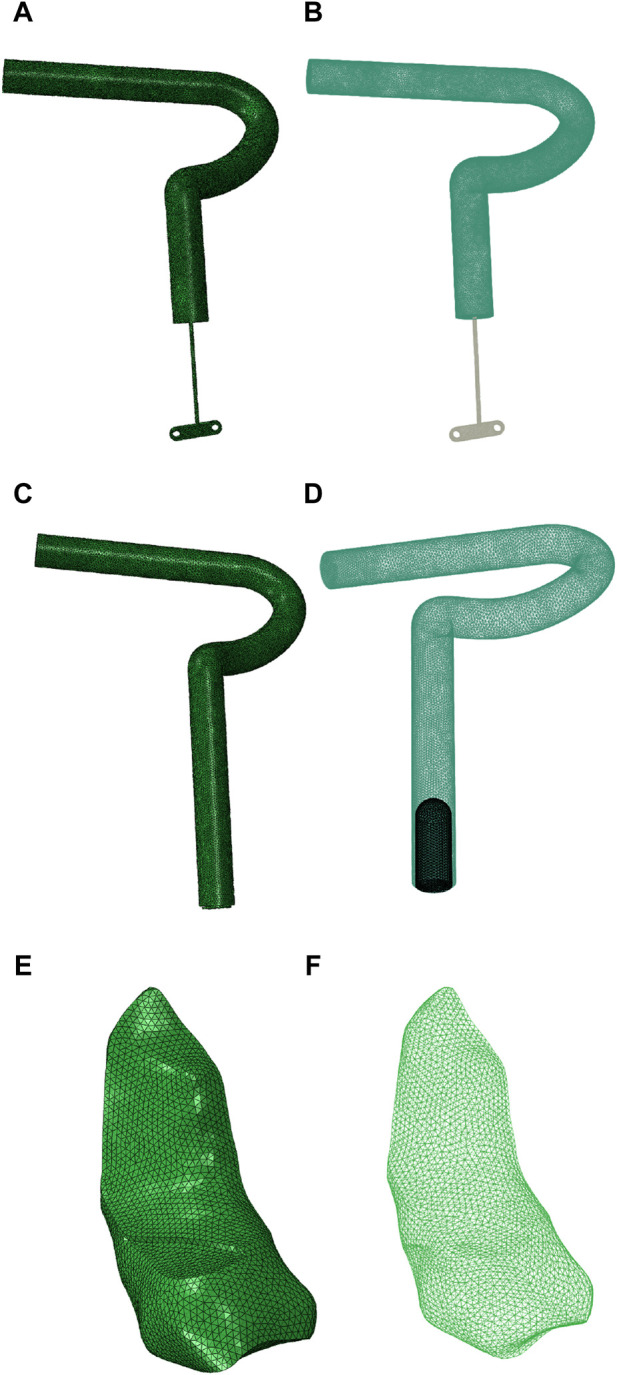
**(A, B)** Model of all-inside ACL reconstruction using a graft (including the plate and the loop, frontal view), **(C, D)** model of traditional ACL reconstruction using a graft (including the interference screw, frontal view), and **(E, F)** a normal ACL model (frontal view).

The graft in the all-inside ACL reconstruction model was shorter than that in the traditional ACL reconstruction model. Its end was connected to the loop and the plate, with the loop diameter set at 1.2 mm and the plate measuring 10 mm in length and 3.2 mm in width, with two holes measuring 1.5 mm diameter each. The tibial tunnel had two diameters: 8 mm (for the ligament) and 3.5 mm (for the loop). In the traditional ACL reconstruction model, the interference screw was set to be embedded in the tibial tunnel at the far end of the graft, with a diameter of 6 mm (refer to Figure 2 for an illustration).

**FIGURE 3:**
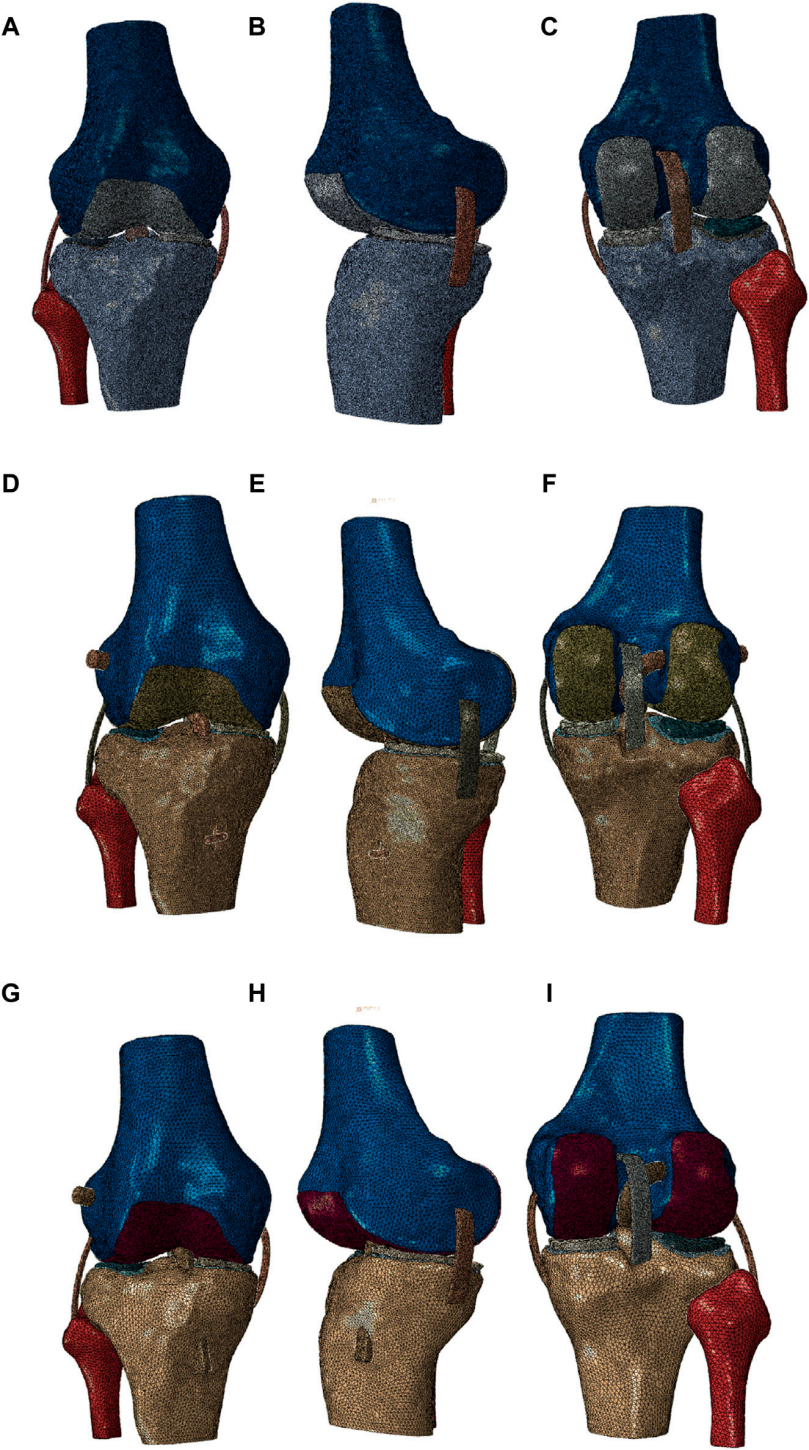
**(A–C)** Frontal view, lateral view, and posterior view of the normal knee joint model, **(D–F)** frontal view, lateral view, and posterior view of the all-inside ACL reconstruction model, and **(G–I)** frontal view, lateral view, and posterior view of the traditional ACL reconstruction model.

In this study, the normal knee joint model included 1,756,479 elements and 427,962 nodes, the all-inside ACL reconstruction model included 1,042,967 elements and 272,150 nodes, and the traditional ACL reconstruction model included 759,036 elements and 215,654 nodes (refer to Figure 3 for visualization).

### Ligament stress calculations for different models

Under different loading conditions, the maximum stress of the ligament in the normal model fluctuated from 1.949 to 18.302 MPa. The maximum stress of the graft in the all-inside ACL reconstruction model fluctuated from 0.705 to 3.465 MPa. In the traditional ACL reconstruction model, the maximum stress of the graft fluctuated from 5.012 to 59.269 MPa. The results of traditional reconstruction of the ACL with a graft in this study were consistent with those in a study by Kim et al. (2021) (6.1–12.3 MPa), demonstrating the reliability of our study results (refer to Table 2 for details).

**TABLE 2:** Measurement of ACL stress under different loading conditions.

Loading condition	Normal group (MPa)	All-inside group (MPa)	Traditional group (MPa)
F = 134 N	1.949	1.303	8.750
External rotation	2.305	1.137	5.012
Internal rotation	2.474	0.705	8.273
Varus	16.686	3.465	5.806
Valgus	18.302	1.672	12.1
Flexion	5.626	1.464	6.656
Extension	16.204	0.992	59.269
Mean (SD)	6.587 (6.891)	1.534 (0.908)	15.124 (19.606)

Abbreviations: normal group, normal model group; all-inside group, the ACL was reconstructed with the all-inside technique; traditional group, the ACL was reconstructed with the traditional technique; mean (SD), mean and standard deviation.

The Mann–Whitney U test revealed that there was no statistically significant difference (*P* = 0.053) between the ligaments of the normal model and the grafts of the all-inside ACL reconstruction model under different loading conditions. There was also no statistically significant difference (*P* = 0.165) between the ligaments of the normal model and the grafts of the traditional ACL reconstruction model under different loading conditions. Additionally, there was a statistically significant difference (*P* = 0.001) between the grafts used in the all-inside ACL reconstruction model and those used in the traditional ACL reconstruction model under different loading conditions (refer to Table 3 for details).

**TABLE 3:** Statistical analysis results of the three models.

Model	U value	P value
Normal group vs. all-inside group	40	0.053
Normal group vs. traditional group	13	0.165
All-inside group vs. traditional group	0	0.001

Abbreviations: normal group, normal model group; all-inside group, the ACL was reconstructed with the all-inside technique; traditional group, the ACL was reconstructed with the traditional technique.

### Ligament stress distribution results for different models

**FIGURE 4:**
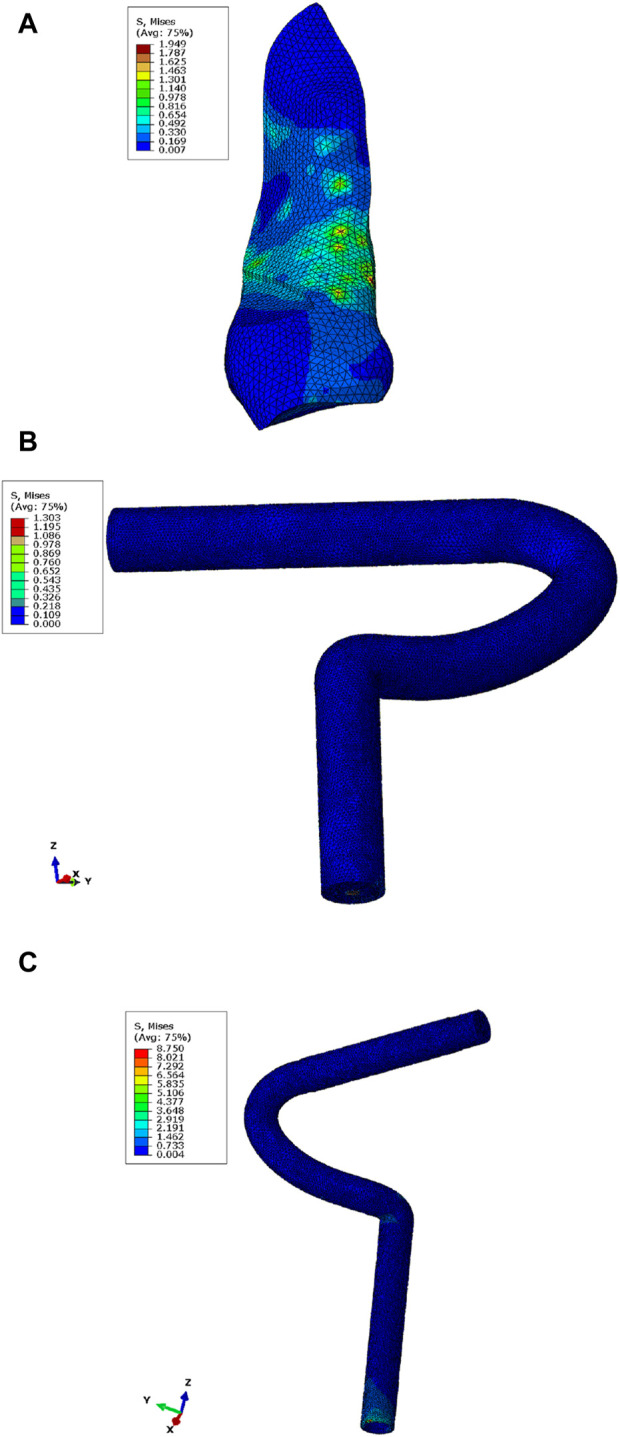
Maximum stress nephogram of the ACL or graft in the models under a force of 134 N applied to the front of the tibia. **(A)** Normal model, with maximum stress distributed on the anterior-medial side of the ligament. **(B)** Graft in the all-inside ACL reconstruction model, with maximum stress distributed at the junction with the loop. **(C)** Graft in the traditional ACL reconstruction model (posterior view), with maximum stress distributed at the junction with the far end of the tibial tunnel.

Under different loading conditions, the maximum stress distribution on the ligaments or grafts in the different models is shown in Figure 4. When a force of 134 N was applied to the front of the tibia in the normal model, the maximum stress of the ligament was mainly distributed on the anterior-medial side. During external rotation, internal rotation, varus, valgus, flexion, and extension movements of the tibia, the maximum stress of the ligament was distributed at the junction with the tibial surface, junction with the tibial surface, lateral middle part, junction with the tibial surface, anterior-medial side, and upper front part of the ligament, respectively.

In the all-inside ACL reconstruction model, when a force of 134 N was applied to the front of the tibia, the maximum stress of the graft was mainly distributed at the junction with the loop. During external rotation, internal rotation, varus, valgus, flexion, and extension movements of the tibia, the maximum stress of the graft was distributed at the junction with the loop. During internal rotational movement of the tibia, the maximum stress of the graft was distributed at the junction with the tibia.

In the traditional ACL reconstruction model, when a force of 134 N was applied to the front of the tibia, the maximum stress of the graft was mainly distributed at the junction with the far end of the tibial tunnel. Similarly, during external rotation, internal rotation, varus, valgus, flexion, and extension movements of the tibia, the maximum stress of the graft was distributed at the junction with the far end of the tibial tunnel.

### Results of the maximum stress of the loop and the plate and the interference screw

The stress distribution differs between the loop and the plate in the all-inside ACL reconstruction model and between the loop and the plate in the traditional ACL reconstruction model. Under different external force conditions, the stress experienced by the loop and the plate in the all-inside ACL reconstruction model ranges from 70.461 to 346.363 MPa. The maximum stress of the interference screw in the traditional ACL model fluctuates from 10.184 to 92.298 MPa. The Mann–Whitney U test revealed a statistically significant difference between the two groups (*P* = 0.002), as shown in Table 4, 5.

**TABLE 4:** Maximum stress of the loop and the plate vs. the interference screw under different external loads.

Load application method	All-inside technique (MPa)	Traditional group (MPa)
F = 134 N	149.384	19.644
External rotation	71.011	10.184
Internal rotation	70.461	11.992
Valgus	346.363	15.944
Varus	308.221	24.020
Flexion	168.034	15.194
Hyperextension	190.285	92.298
Mean (SD)	186.251 (107.166)	27.039 (29.145)

Abbreviations: mean (SD), mean and standard deviation.

**TABLE 5:** Loop, plate, and interference screw results.

Model	U value	*P* value
All-inside vs. traditional	47	0.002

**FIGURE 5:**
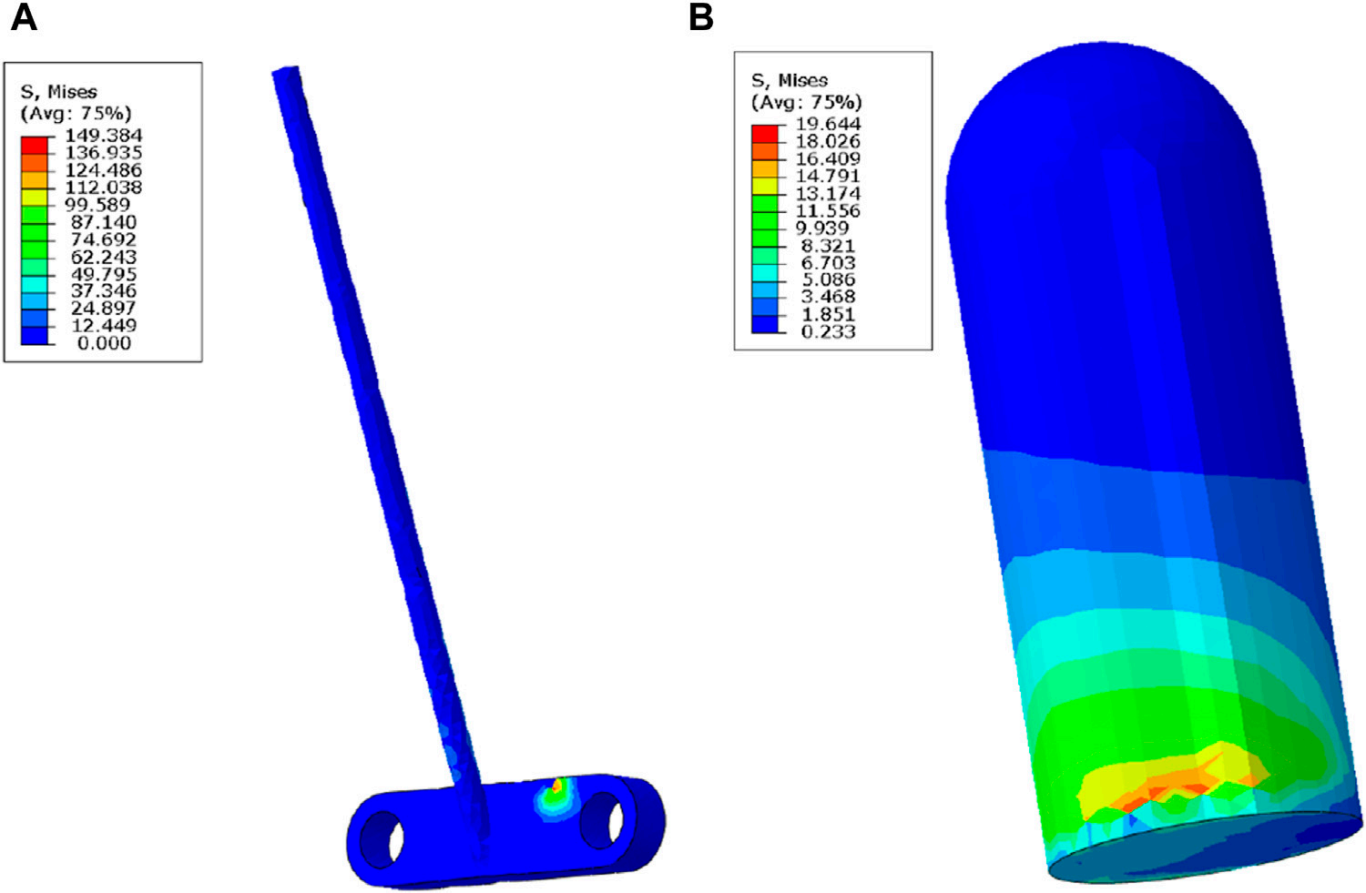
Maximum stress nephogram of the loop and the plate in the all-inside ACL reconstruction model (straight line portion), with maximum stress distributed on the plate at the interface with the tibia **(A)** and at the interference screw in the traditional ACL reconstruction model, with maximum stress distributed at the distal end of the interference screw at the interface with the ligament **(B)**.

Under different external load conditions, the maximum stress of the loop and the plate in the all-inside ACL reconstruction model was primarily distributed on the plate and at the interface with the tibia. Similarly, the maximum stress of the interference screw in the traditional ACL reconstruction model was mainly distributed at the distal end of the screw. This distribution is illustrated in Figure 5.

## Discussion

Various possible activities of the knee joint under forward external force and physiological conditions were simulated in this study. The results suggest that under the same external load, the graft used in all-inside ACL reconstruction was exposed to less stress than that used in traditional ACL reconstruction. The difference was statistically significant (*P* = 0.001). Additionally, the graft used in the all-inside ACL reconstruction model was exposed to less stress than that used in the normal model under similar mechanical conditions (*P* = 0.053), which were consistent with the findings of other studies (Wang et al., 2020). It was further verified that compared with traditional ACL reconstruction, all-inside ACL reconstruction has advantages in terms of mechanical properties.

During knee joint hyperextension, the stress on the graft used in traditional ACL reconstruction rapidly increases to 59.296 MPa, indicating significant stress changes relative to other models and potentially indicating a higher risk of damage with traditional ACL reconstruction. Studies have revealed a correlation between excessive graft stress and postoperative pain and early surgical failure (Bachmaier et al., 2023). This may suggest that all-inside ACL reconstruction offers better postoperative graft stability, potentially leading to better patient outcomes under such mechanical conditions.

The distribution stress on normal ligaments is unknown under different external force conditions. In contrast, the grafts used for reconstruction exhibit relatively fixed stress distribution patterns under various movements. In all-inside ACL reconstruction, stress on the graft is maximally concentrated at the junction of the graft and the loop. In traditional ACL reconstruction, stress is primarily concentrated at the junction of the interference screw and the graft. This study revealed that in all-inside ACL reconstruction, attention should be given to ensuring stability at the junction of the graft and the loop, mainly by ensuring that the strength of the loop meets the requirements. Thus, potential weaknesses of this technique may lie in this area. Future improvements to surgery may focus on dispersing stress at this junction to avoid adverse outcomes.

During mechanical analysis of the loop, plate, and interference screw, it was observed that the stress on the loop and plate was greater than that on the traditional interference screw. One possible reason is that different materials were used in this study (a titanium alloy loop and plate with a Young’s modulus of 1,10,000 MPa and an interference screw with a Young’s modulus of 1,200 MPa). Even though there was a considerable amount of stress on the loop and plate (up to 346.363 MPa), it still did not equal the yield stress of titanium alloys (ranging from 800 to 1,000 MPa) (Ghouse et al., 2018; Huang et al., 2023). This indicates that all-inside ACL reconstruction can withstand the stresses generated by daily knee joint activities to some extent.

The main differences between all-inside ACL reconstruction and traditional ACL reconstruction are as follows. First, the autogenous ligament used for all-inside ACL reconstruction is shorter than that used for traditional reconstruction. Second, traditional reconstruction involves drilling from the tibial end to create the ligament tunnel, whereas all-inside reconstruction involves drilling approximately halfway into the knee joint at the tibial end (diameter approximately 8 mm) and then switching to a smaller drill (diameter approximately 3.5 mm) to drill through the tibial cortex, followed by suspending the ligament on the tibial surface using the plate. In all-inside ACL reconstruction, a flipcutter technique was used for bilateral cortical bone fixation, which provides stronger and minimally invasive fixation. Third, due to the smaller incision used for all-inside ACL reconstruction, the postoperative recovery time is shorter. Fourth, the all-inside technique preserves more of the tibial bone and causes less damage to the bone, resulting in lower risks of postoperative bleeding and infection, which is beneficial for postoperative bone healing (Yang et al., 2022; Takahashi et al., 2023; Li et al., 2024).

The all-inside ACL reconstruction technique was first proposed by Morgan et al. in 1995 (Liu et al., 2023) and then further improved by Lubowitz et al. (2011). The technique is now widely used in clinical practice. The research by Bosco et al. (2023) showed that the all-inside ACL reconstruction technique can be applied to all ACL injuries and open epiphyses in adolescents. Bora and Deshmukh (2023) conducted a study on 324 patients who underwent all-inside ACL reconstruction and reported the results of the knee joint pain assessment, stiffness test, and Lachman test before surgery and at 3, 6, and 12 months postoperatively. All the measurements of the observation indicators significantly improved in the study. They believe that orthopedic surgeons can consider this technique an effective alternative to traditional surgical methods as it shortens the total recovery time for patients, allowing them to recover movement more quickly. This conclusion has been confirmed by Li et al. (2024). A long-term follow-up study (Pichler et al., 2023) showed no significant difference in knee joint function prognosis between male and female athletes who underwent all-inside ACL reconstruction.

This study has several limitations. First, the ligament properties set in this study were isotropic material properties, whereas the actual ligament structure was mainly composed of a matrix and fibers with different mechanical property parameters (Salehghaffari and Dhaher, 2015; Adouni et al., 2020; Cheng et al., 2022). Therefore, the research results may deviate from actual clinical outcomes. Second, the study overlooked the patella, patellar ligament, joint fluid, and other soft tissues around the knee joint, which may have led to discrepancies between the study results and the actual knee joint movements. In addition, the role of the patella and its patellar ligament in stabilizing the knee joint was not investigated in this study, which may further affect the research results (Dhaher et al., 2016). Third, this study is limited to the local aspects of the knee joint and does not fully model the effects of the femur, tibia, and fibula on the graft during knee joint movements. Therefore, in future studies, it may be necessary to establish a model that includes a complete femur, tibia, fibula, and patella, and simultaneously considers the properties of the surrounding soft tissue materials and select a suitable hyperelastic material model, which may be more realistic.

## Conclusion

In summary, under different external forces, the all-inside ACL reconstruction results in less stress on the graft than does traditional ACL reconstruction. This may suggest a relatively stable mechanical environment, indicating that the strength of the plate theoretically reflects the daily knee joint movements of patients without injury.

## Data Availability

The datasets presented in this article are not readily available because the content of our research data is confidential. If we share it, the technical handling skills of our research process will be exposed. Requests to access the datasets should be directed to nothing.

## Abbreviations

ACL, anterior cruciate ligament; CT, computed tomography.
